# ILF2 Directly Binds and Stabilizes CREB to Stimulate Malignant Phenotypes of Liver Cancer Cells

**DOI:** 10.1155/2019/1575031

**Published:** 2019-02-10

**Authors:** Hui Du, Yun Le, Fenyong Sun, Kai Li, Yanfeng Xu

**Affiliations:** ^1^Department of Experiment Center, Shanghai Municipal Hospital of Traditional Chinese Medicine, Shanghai University of Traditional Chinese Medicine, Shanghai 200071, China; ^2^Department of Pharmacy, Changhai Hospital of Shanghai, 200433, China; ^3^Department of Clinical Laboratory Medicine, Shanghai Tenth People's Hospital of Tongji University, Shanghai 200072, China; ^4^People's Hospital of Pudong New District of Shanghai City, No. 490, South Chuanhuan Road, Shanghai 201200, China; ^5^Department of Pharmacy, Shanghai Municipal Hospital of Traditional Chinese Medicine, Shanghai University of Traditional Chinese Medicine, Shanghai 200071, China

## Abstract

Cyclic adenosine monophosphate (cAMP) response element-binding protein (CREB) is overexpressed and has an oncogenic role in hepatocellular carcinoma (HCC). Interleukin enhancer binding factor 2 (ILF2) has become research hotspot in liver cancer recently. However, it is still unclear whether and how CREB and ILF2 interact with each other. And how this interaction exerts its role in occurrence and development of liver cancer is still unclear. Here, we found that ILF2 directly bound with CREB, and this binding was essential for the malignant phenotypes of liver cancer cells. Moreover, we found that ILF2 acted as one of the upstream proteins of CREB and promoted CREB only in the protein level, whereas ILF2 expression was not regulated by CREB. Mechanistically, ILF2 bound to the pKID domain of CREB and stimulated its phosphorylation at Ser133. Taken together, our study finds a novel interaction between CREB and ILF2 in liver cancer, and this interaction might play a role in the diagnosis and remedy of liver cancer.

## 1. Introduction

Liver cancer is the fifth most common cancer in the world and the third leading cause of cancer deaths [1]. Although considerable progress has been made in treating patients with liver cancer, surgical resection remains the preferred option for these patients, with an approximate 30% to 40% 5-year survival rate [2]. The unclear pathogenesis of liver cancer results in the limited treatment options for its patients. Recently, CREB protein has been linked to liver cancer [3]. As a transcription factor, CREB exerts its role via binding the promoter containing CRE motif. Long noncoding RNA, HOTAIR, increases the growth of human liver cancer stem cells by downregulating SETD2 via reducing its recruitment of CREB [4]. Moreover, the mechanical abnormal pain and spontaneous pain caused by bone cancer could be alleviated when the positive feedback regulation between CREB/CRTC1 and its target gene miR-132 was interdicted [5]. A large number of researches have shown that many types of cancer cells grow slowly when CREB is knocked out [6–9]. Since CREB is indispensable for the development of liver cancer, investigating how CREB plays its carcinogenic role in liver cancer is extremely important.

ILF2 is one type of transcription factor and is also named as the nuclear factor of activated T cells (NF45). It regulates cell growth via regulating mRNA stability. Increased mRNA levels of NF45 were observed in lymphoma and leukemia cell lines [10]. Liver cancer cells would grow rapidly when infected with ILF2 overexpression plasmids [11]. Recent evidences indicate that ILF2 is highly expressed in non-small-cell lung cancer, glioma, lymphoma, leukemia, and cervical cancer, and high expression of ILF2 is associated with poor clinical outcome [12–15]. Subcellular tissue proteomics also regards ILF2 as a molecular stimulus of liver cancer [16]. These findings suggest that ILF2 might play a critical role in regulating cancer cell growth. However, the exact mechanism of ILF2 in liver cancer is almost unknown.

In the current work, we emphasized the importance of CREB and ILF2 in stimulating malignant phenotypes of liver cancer cells. Our work also suggested that these two proteins had a positive relationship in tumor tissues, indicating that they might play key roles in liver cancer. In summary, our work summarizes a new relationship between these two proteins and uncovers the mechanism between them.

## 2. Material and Methods

### 2.1. Cell Culture, Vectors, and Tissue Samples

HEK-293T, Bel-7402, and SMMC-7721 cells obtained from the cell bank of the Chinese Academy of Sciences (Shanghai, China) were cultured in Dulbecco's modified Eagle's medium (DMEM) (HyClone, Thermo Scientific, San Jose, CA, USA) supplemented with 10% fetal bovine serum (FBS) (Gibco-BRL, Invitrogen Life Technologies, Carlsbad, CA, USA) and were incubated in 37°C and 5% CO_2_. Cells were treated with cycloheximide (CHX) (Sigma, St. Louis, MO, USA) at a final concentration of 50 *μ*g/ml. Tumorous and normal liver tissues were gained at Shanghai Tenth People's Hospital under institutional approval. Informed written consent in the study was gained from all participants. The short hairpin RNAs (shRNAs) against human CREB (hCREB) (sh1&sh2) (targeting CDS sequence), lentiviral-based hCREB-HA, and lentiviral-based human ILF2- (hILF2-) FLAG-expressing plasmids were purchased from Biolink (Shanghai, China). The shRNAs against hILF2 (sh1&sh2) (targeting 3'UTR sequence) were bought from GeneChem (Shanghai, China). Plasmids were transfected using Lipofectamine 2000 reagent following the manufacturer's instructions. WT-CREB, Del-pKID-CREB, and Del-bZIP-CREB were constructed by overlapping PCR using pcDNA3.1(+) as the backbone. Primers used were WT-CREB-F: ACTGGAATTCATGACCATGGAATCTGGAGCCGAGAACC and WT-CREB-R: ACTGGATATCTTAAGCGTAGTCTGGGACGTCGTATGGGTAATCTGATTTGTGGCAGTAAAGGTC; Del-pKID-CREB-F: GAAGAAGAGAAGTCTGAAGAGGAGA and DEL-PKID-CREB-R: AGACTTCTCTTCTTCCTGTGAATCTTCACTTTCTGCAATA; Del-bZIP-CREB-F: ACTGGAATTCATGACCATGGAATCTGGAGCCGAGAACC and Del-bZIP-CREB-R: ACTGGATATCTTAAGCGTAGTCTGGGACGTCGTATGGGTAATCTGATCGTGCTGCTTCTTCAGCAG.

### 2.2. Immunohistochemistry (IHC), Western Blotting (WB), and Immunofluorescence (IF)

For IHC, tissue sections were deparaffinized and hydrated in xylene and serial alcohol solutions, respectively. Endogenous peroxidase activity was blocked by incubation in sodium citrate at 100°C for 2 hours. The specimen was blocked by 5% normal goat serum at room temperature for one hour before being incubated with primary antibodies anti-CREB (Cell Signaling Technology (CST), Boston, MA, USA, #9197) or anti-ILF2 (Abcam, Hong Kong, China, #ab154169) overnight at 4°C. The slices were incubated with secondary antibody signal stain (R) boost IHC detection reagent (HRP, mouse/rabbit) at room temperature for one hour. Antigen-antibody reactions were detected with DAB (Vector Lab, America) under the instructions. The stain was counterstained with hematoxylin, dehydrated in ethanol, cleared in xylene, coverslipped, and photographed by Leica camera.

For WB, the cells were lysed in western/IP lysis buffer (Beyotime, Haimen, China, #P0013) on ice for 30 minutes and centrifuged at 15,000 g for 15 minutes at 4°C to collect whole cell lysates. Protein extracts were quantitated with BCA protein assay kit (Pierce Biotechnology, Rockford, IL, USA), subjected to electrophoresis on a 10% SDS-PAGE gel with 100–200 V, and transferred onto a nitrocellulose filter membrane (Roche, Penzberg, Germany) at 200 mA with cooling. The membrane was blocked with 5% nonfat milk dissolved in Tris-buffered saline Tween-20 (PBST) at room temperature for one hour. Membranes were incubated with anti-CREB (CST, #9197), anti-p-CREB (CST, #9198), anti-ILF2 (Abcam, #ab154169), or anti-GAPDH (CST, #5174) overnight at 4°C, washed with PBST for 10 minutes three times, and then incubated with anti-rabbit IgG, HRP-linked antibody (CST, #7071) at room temperature for one hour. After washing, protein bands were visualized using enhanced chemiluminescence (ECL) coloring fluid, developer and fixer. The relative expression levels of proteins were determined by densitometry using ImageJ software and were normalized against GAPDH.

For IF on cell cover slips, cells were seeded on the cover slips in 24-well cell culture plates, fixed with 4% paraformaldehyde (PFA) for 15 minutes after 24 hours, washed with PBS for 5 minutes three times, permeabilized with 1% Triton X-100/PBS for 15 minutes, blocked by 1% BSA for 30 minutes, then incubated with the primary antibodies anti-CREB (CST, #9104) or anti-ILF2 (Abcam, #ab154169) overnight at 4°C. After washing, the cells were incubated with the secondary antibodies fluorescent Alexa Fluor 488 or Alexa Fluor 555 (Life Technologies, Carlsbad, CA, USA) at room temperature for one hour and counterstained with DAPI (Invitrogen, America). Images were obtained by Leica DMRA fluorescence microscope. For IF on tissue sections, after the antigen was repaired, the remaining steps were the same as those on the cell cover slips.

### 2.3. Coimmunoprecipitation (co-IP)

co-IP was performed as described previously [17]. Briefly, the cell lysate was incubated with protein A/G-Sepharose (Novex, Oslo, Norway) and 3 *μ*l antibodies, shook overnight at 4°C, washed with western/IP lysis buffer, and centrifuged at 3500 rpm for 1 minute at room temperature three times. The remaining steps were similar to western blotting. The antibodies used for IP were anti-CREB (CST, #9104), anti-CREB (CST, #9197), anti-ILF2 (Abcam, #ab154169), anti-FLAG (CST, #8146), and anti-HA (CST, #2367). For *in vitro* reciprocal co-IP, the reagents used were purified protein CREB (Abnova, Taiwan, #H00001385-P01), protein ILF2 (Abnova, #H00003608-P01), and DTT (Beyotime, #ST041) and the method has been described before [18, 19].

### 2.4. Cell Proliferation, Soft Agar Colony Formation, Caspase 3/7 Activity, and Quantitative RT-PCR (qPCR)

Cell proliferation, caspase 3/7 activity, and colony formation capacity were measured using MTT-based assay, caspase 3/7 Glo luciferase reagent (Promega, Madison, USA), and soft agar colony formation assay, respectively, which were described previously [20]. The total RNA from the cells was obtained by using TRIzol (TransGen Biotech, Beijing, China), reversed into cDNA with the help of PrimeScript RT Master Mix Kit (Takara, Japan). qPCR was performed in Applied Biosystems 7500 Real-Time PCR System (Life Technologies) with KAPA SYBR FAST qPCR Kit Master Mix (KAPA Biosystems). The human GAPDH gene was used as an internal control. The primers used in the qPCR were GAPDH-F: 5′CCATCTTCCAGGAGCGAGATCCCTCC 3′ and GAPDH-R: 5′GGTGCAGGAGGCATTGCTGATGATC 3′; CREB-F: 5′GCCCAGGTATCTATGCCAGCAGCTC 3′ and CREB-R: 5′CAAAATTTTCCTGTAGGAAGGCCTCC 3′; MCAM-F: 5′GCGTCTACAAAGCTCCGGAGGA 3′ and MCAM-R: 5′GAATGTGGACCCGGTTCTTCTCCTC 3′; HULC-F: 5′ACCTCCAGAACTGTGATCCAAAATG 3′ and HULC-R: 5′CAAATTTGCCACAGGTTGAACAC 3′; ILF2-F: GGAAGCTGTTGCTGCCCTGGGGAAC and ILF2-R: GCAATACTTTGATATCCAAATGG.

### 2.5. Protein Ligation Assay (PLA)

The protein ligation assay was carried out to identify the direct interaction between CREB and ILF2 or ILF3 using the Duolink™ in situ red starter kit (mouse/rabbit) (Sigma, Uppsala, Sweden). The cells were seeded on glass cover slips in 24-well plates. On the second day, the cells were fixed with 4% PFA for 15 min and blocked with the blocking buffer supplied by the manufacturer for 1 h. After blocking, the cells were incubated overnight at 4°C with the indicated suitable primary antibodies. The primary antibodies used were anti-CREB (CST, #9197), anti-ILF2 (Abcam, #ab154169), and anti-ILF3 (Abcam, #ab225619). On the third day, the PLA probe solution (supplied by the manufacturer, Sigma) was added into each well for 1 h at 37°C, and the ligase-ligase solution (supplied by the manufacturer) was added into each well and incubated for 30 min at 37°C. After ligation, the amplification-polymerase solution (supplied by the manufacturer) was added into each well for another 100 min at 37°C and subjected to microscopic analysis. When the proximity of two PLA probes is less than 40 nm, the red fluorescent emissions can be detected [21].

### 2.6. Mass Spectrometry (MS) Analysis

The specific method was described before [22]. The partial work was completed in Shanghai Jiao Tong University. The instruments used were LC system (Nano Pump, Ultimate 3000, Dionex, Thermo Fisher) and ESI-Q-TOF mass spectrometer (maXis, Impact, Bruker Daltonik, Germany). The mass spectrometry proteomics data have been deposited to the ProteomeXchange Consortium via the PRIDE. Data are available via ProteomeXchange with identifier PXD008261.

### 2.7. Statistical Analysis

Data were analyzed using SPSS 20.0. Tests used to examine the differences between groups included the Student *t*-test, one-way ANOVA test, and the Spearman rank-correlation analysis. *P* < 0.05 was considered statistically significant.

## 3. Results

### 3.1. ILF2 Was Identified to Directly Interact with CREB

**Figure 1:**
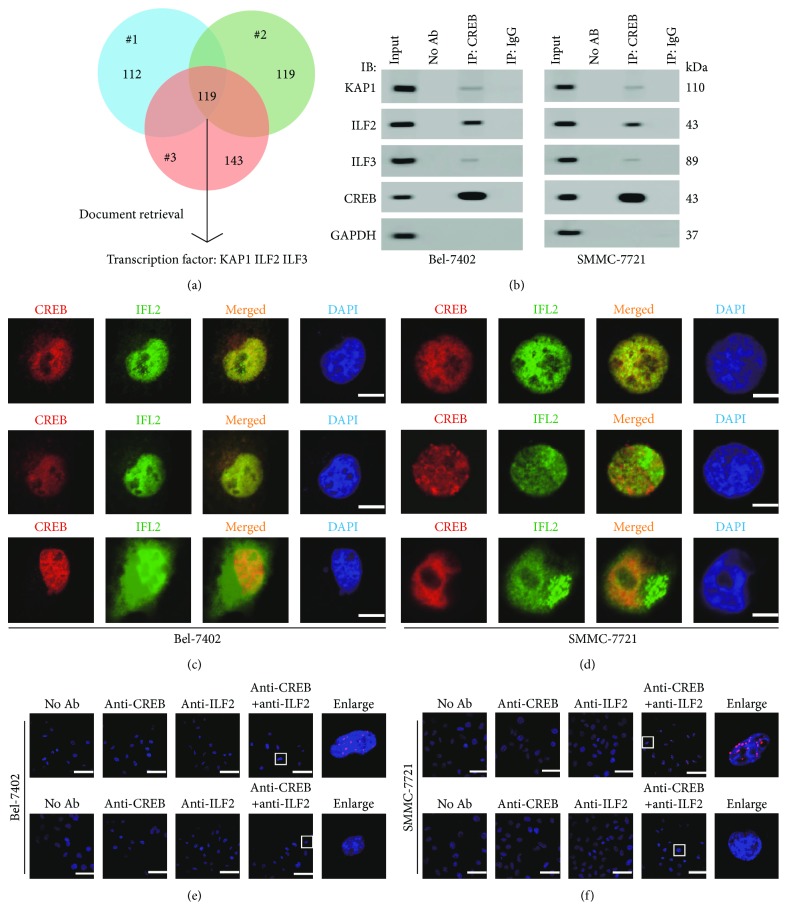
ILF2 was identified to directly interact with CREB. (a) MS analysis of CREB-binding proteins. The proteins immunoprecipitated by anti-CREB antibodies were analyzed by ESI-Q-TOF-MS in Bel-7402 cells for three independent times. (b) Endogenous CREB was immunoprecipitated by anti-CREB antibodies in Bel-7402 and SMMC-7721 cells, and the indicated proteins were measured by WB. The negative control for IP was immunoglobulin G (IgG). (c, d) The colocalization of CREB and other transcription factors in Bel-7402 and SMMC-7721. The nuclei were stained with DAPI. Representative images of three independent experiments were shown. Cells were obtained for IF by anti-CREB and corresponding antibodies. Scale bar, 15 *μ*m. (e, f) The interactions between CREB and ILF2 or ILF3 were analyzed by PLA. The rightmost graphic was the enlargements of the boxes. Scale bar, 50 *μ*m.

Proteins pulled down by anti-CREB antibody were analyzed by the mass spectrometry analysis, and three transcription factors KAP1, ILF2, and ILF3 were identified via literature retrieval [11, 23, 24] (Figure 1(a)). Then the co-IP experiments were performed and we found that endogenous KAP1, ILF2, and ILF3 proteins could be coimmunoprecipitated by anti-CREB antibodies in Bel-7402 and SMMC-7721 cells, and the binding of ILF2 to CREB was stronger compared to that of the other two proteins (Figure 1(b)). Moreover, endogenous CREB could be coimmunoprecipitated by anti-ILF2 antibodies (Supplementary Figure 1). To understand the localization of these proteins, we performed IF and found that CREB, ILF2, and ILF3 were all cell nuclear localized in Bel-7402 and SMMC-7721 cells. However, KAP1 localized both in nuclear and in the cytoplasm (Figures 1(c) and 1(d)), indicating that the distance between CREB and KAP1 might be much longer than the distance between CREB and ILF2 or ILF3. Therefore, we excluded KAP1 protein as a direct CREB interactor. The exact direct interactions between CREB and ILF2 or ILF3 were determined by PLA (Figures 1(e) and 1(f)). The red spots were only found in the ILF2 group, suggesting that ILF2, instead of ILF3, was a direct CREB interactor.

### 3.2. CREB Interacts with ILF2 in Liver Cancer

**Figure 2:**
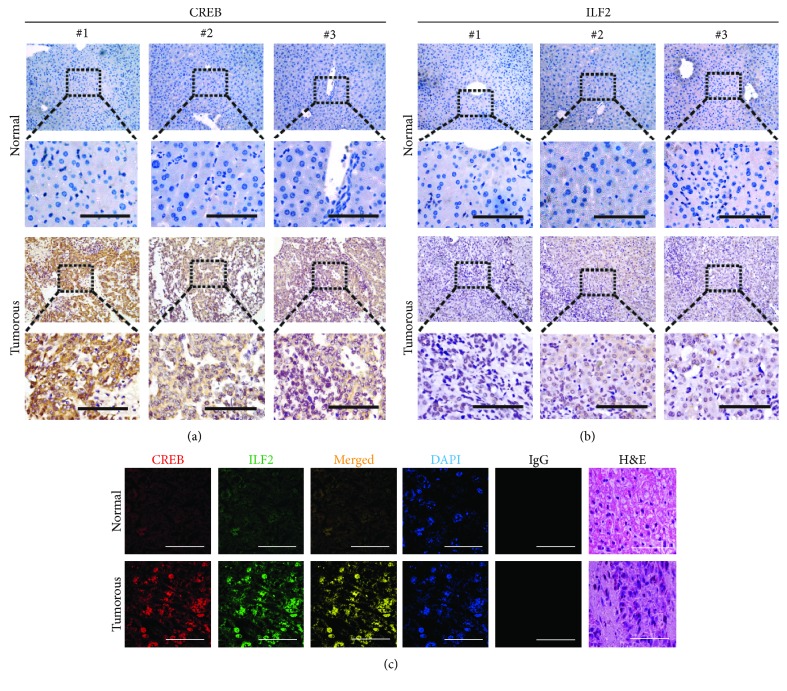
CREB interacts with ILF2 in liver cancer. (a, b) IHC staining of CREB and ILF2 expression in liver cancer tissues and adjacent noncancerous tissues. Scale bar, 500 *μ*m. (c) The subcellular localization of CREB and ILF2 in liver cancer tissues and adjacent normal liver tissues was measured by IF. Representative images of three independent experiments were shown. The rightmost graphic was the H&E staining of tissue sections. Scale bar, 15 *μ*m.

To understand the expression of two proteins in liver tissues, we detected mRNA levels of CREB and ILF2 and observed that CREB and ILF2 mRNA levels were upregulated in liver cancer tissues compared to adjacent normal tissues (Supplementary Figure 2(a)). A positive correlation was identified for the fold change (tumorous vs normal) between CREB and ILF2 (*R* = 0.819, *P* < 0.001) (Supplementary Figure 2(b)). Moreover, increased protein expressions of CREB and ILF2 were found in liver cancer tissues compared to adjacent normal tissues (Supplementary Figure 2(c)). Furthermore, we performed IHC experiments using three paired liver cancer and adjacent noncancerous tissues. The phenomenon of high expression of CREB and ILF2 proteins in liver cancer tissues compared to adjacent normal liver tissues was also observed (Figures 2(a) and 2(b) and Supplementary Figure 2(d)). Then we performed IF using tissues and found that expressions of CREB and ILF2 were stronger in tumorous tissues compared to adjacent normal tissues (Figure 2(c)). In order to further verify the interaction between CREB and ILF2, we conducted reciprocal co-IP experiments and observed that exogenous ILF2-FLAG could be easily pulled down by CREB-HA and *vice versa* (Supplementary Figure 2(e)). Moreover, we confirmed that the two proteins could interact directly with each other by the co-IP experiment using purified CREB and ILF2 proteins *in vitro* (Supplementary Figure 2(f)). These results further suggested that CREB directly interacts with ILF2, and this interaction is clinically common.

### 3.3. ILF2 Is an Upstream Protein of CREB

**Figure 3:**
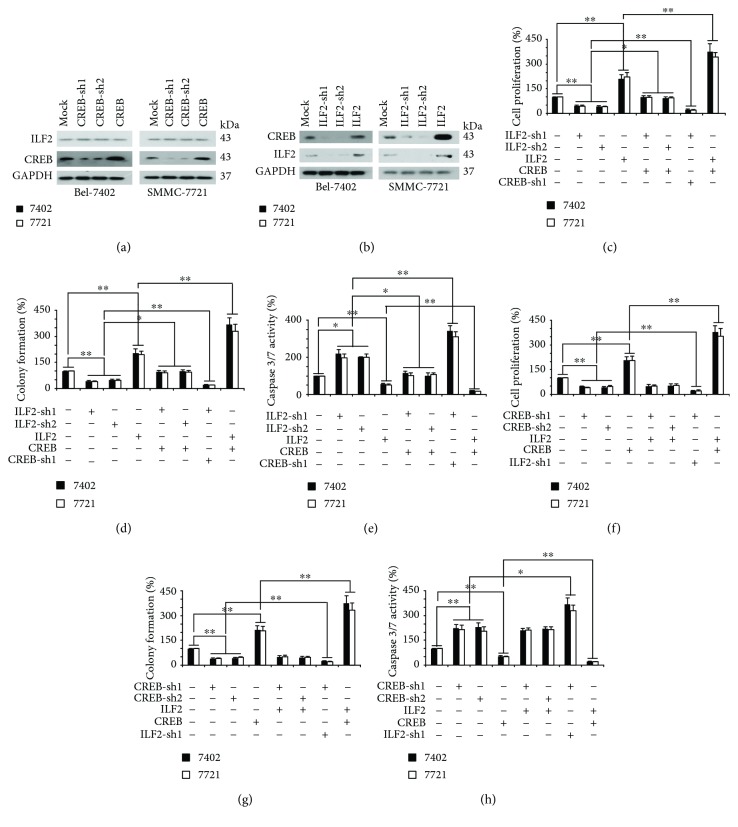
ILF2 is an upstream protein of CREB. (a, b) The ILF2, CREB, and GAPDH protein expression levels in control (infected with shRNA against GFP), Bel-7402, or SMMC-7721 cells with knockdown and overexpression of CREB or ILF2 were measured by western blotting. (c–e) Cell proliferation, colony formation capacity, and apoptosis status in control cells and Bel-7402 or SMMC-7721 cells with indicated plasmids transfected. (f–h) Cell proliferation, colony formation capacity, and apoptosis status in control cells and Bel-7402 or SMMC-7721 cells with indicated plasmids transfected. Data were exhibited as mean ± SD of three independent experiments. ^∗^ *P* < 0.05, ^∗∗^ *P* < 0.01 indicates statistical significance. Data were analyzed using the Student *t-*test.

We observed that overexpression or knockdown of CREB did not regulate the expression of ILF2, while knockdown of ILF2 resulted in a significant reduction of CREB in both Bel-7402 and SMMC-7721 cells (Figures 3(a) and 3(b)). Then we tested whether ILF2 and CREB are essential in the process of liver cancer development. In order to reduce the expression of ILF2 and CREB in two liver cancer cells, ILF2- or CREB-specific shRNAs were used. We found that the ability of cell proliferation was weakened in two cell lines when either ILF2 or CREB was inhibited (Figures 3(c) and 3(f)). In addition, the weakened ability of colony formation was observed with the knockdown of ILF2 or CREB (Figures 3(d) and 3(g) and Supplementary Figures 3(a) and 3(b)). However, increased apoptosis could be tested while using these shRNAs, measured by caspase 3/7 Glo luciferase reagent (Figures 3(e) and 3(h)). In contrast, overexpression of the two proteins had opposite effects. Furthermore, we found that changes in cell function by shRNAs against ILF2 could be rescued by overexpression of CREB at the same time (Figures 3(c)–3(e)). But simultaneous ectopic expression of ILF2 could not rescue effects made by shRNAs against CREB (Figures 3(f)–3(h)). These data indicated that ILF2 is an upstream protein of CREB.

We also observed that compared to separately knocked-down or overexpressed ILF2 and CREB, simultaneously knocked-down or overexpressed ILF2 and CREB more obviously inhibited or stimulated the proliferation and colony formation, whereas they stimulated or inhibited the caspase 3/7 activity of liver cancer cells (Figures 3(c)–3(h)). Moreover, expression of CREB was positively associated with the level of cell proliferation and colony formation and negatively associated with the level of caspase 3/7 activity, whereas the expression level of ILF2 was not associated with the level of cell proliferation, colony formation, and caspase 3/7 activity (Figures 3(c)–3(h)) and Supplementary Figures 3(c)–3(d)). These data further demonstrated that ILF2 acts on CREB to stimulate malignant phenotypes of liver cancer cells.

### 3.4. ILF2 Enhances CREB Protein Stability via Inhibiting Its Phosphorylation at Ser133

**Figure 4:**
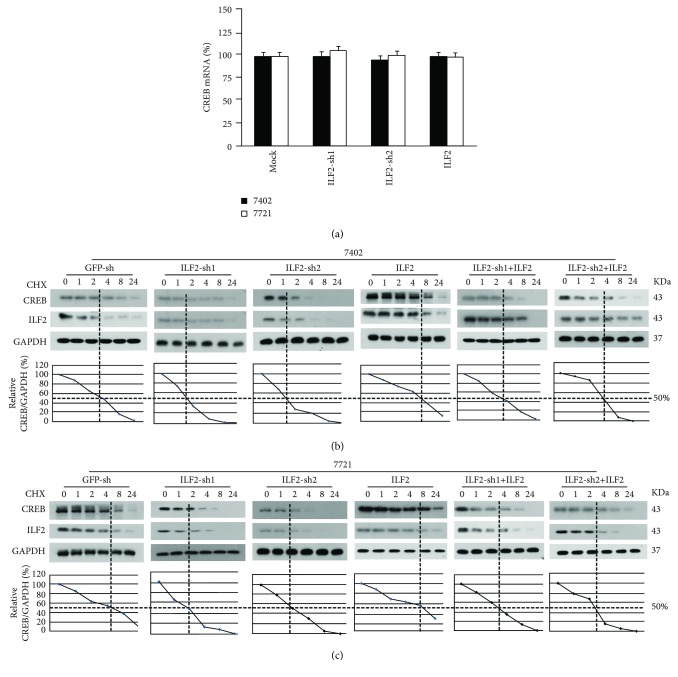
ILF2 enhances CREB protein stability. (a) The mRNA level of CREB was tested by qPCR in control and Bel-7402 or SMMC-7721 cells with knockdown and overexpression of ILF2. (b, c) CHX chase experiments were performed in control and Bel-7402 or SMMC-7721 cells with ILF2 knockdown, ILF2 overexpression, and ILF2 knockdown and ILF2 overexpression simultaneously. The images of ILF2, CREB, and GAPDH were performed by western blotting. The ratios between CREB and GAPDH were calculated with the 0 h point arbitrarily set to 100%.

We subsequently investigated how ILF2 regulates CREB expression. We eliminated the possibility that ILF2 could regulate the mRNA level of CREB by qPCR experiments (Figure 4(a)). In addition, through CHX chase experiments, we found that the half-life of CREB protein could be prolonged by overexpression of ILF2, whereas knockdown of ILF2 promoted CREB degradation. Furthermore, the shortened half-life of CREB protein caused by the knockdown of ILF2 could be rescued by overexpression of exogenous ILF2 in the meantime (Figures 4(b) and 4(c)). The CREB target genes, MCAM [25] and HULC [26], were also downregulated by knockdown of ILF2 and could be rescued by overexpression of exogenous ILF2 at the same time (Supplementary Figures 4(a) and 4(b)). All these data suggested that ILF2 enhances CREB stability without modulating the mRNA level of CREB.

CREB protein contains two critical function domains, pKID domain and bZIP domain [27, 28]. We constructed plasmids with pKID or bZIP domain deleted (Supplementary Figure 4(c)) and observed that pKID domain was essential for the interaction between CREB and ILF2 (Supplementary Figures 4(d)–4(e)). Phosphorylation at Ser133 of CREB significantly inhibits its activity and leads to its degradation [29], and Ser133 coincidentally locates in the pKID domain. WB results indicated that knockdown of ILF2 activated the phosphorylation at Ser133 of CREB, whereas overexpression of ILF2 suppressed the phosphorylation at Ser133 of CREB (Supplementary Figure 4(f)). Therefore, we speculated that ILF2 stabilized CREB probably via inhibiting its phosphorylation at Ser133.

## 4. Discussion

If not diagnosed early, the treatment of liver cancer patients would be very difficult. Despite decades of researches on its etiology and pathogenesis, our understanding of the molecular mechanisms of liver cancer remains superficial. Although we have reported the interplay of YAP and CREB in liver cancer [29], the interaction between CREB and other proteins is still poorly understood. There are increasing evidences suggesting that ILF2 is highly expressed in various types of cancer [12–15]. In this study, we identified the direct interaction between CREB and ILF2. ILF2 acted as the upstream regulator of CREB and positively regulated its stability. Moreover, this interaction widely existed in the clinical liver cancer samples. Targeting this ILF2-CREB interaction might provide new strategies for liver cancer treatment.

In this study, we observed that CREB expression was positively associated with the malignant phenotypes of liver cancer cells, whereas ILF2 expression was not associated with the malignant phenotypes of liver cancer cells. Previous studies have reported that CREB stimulates the expressions of several cancer promoters like YAP [29], HULC [26], c-FLIP(L), and MKP-1 [30]. These downstream effectors all have the function of proliferation promoting or antiapoptosis. Therefore, CREB positively regulates malignant phenotypes of liver cancer cells through its extensive downstream networks. As for ILF2, its direct downstream stimulators in liver cancer have not been reported until now. Therefore, ILF2 acts as a liver cancer stimulator mainly through its regulation on CREB. If CREB is inhibited in liver cancer, ILF2 would lose its ability as a liver cancer stimulator.

We observed that pKID domain, which can play a transcriptional regulatory role and bind to other protein domains [28], played essential roles for the interaction between CREB and ILF2. Furthermore, ILF2 directly inhibited the phosphorylation at Ser 133 in the pKID domain. Phosphorylation at Ser 133 of CREB significantly suppressed its activity. This phosphorylation is catalyzed by MAPK14/p38 and ultimately leads to the CREB degradation which is led by beta-transducin repeat containing E3 ubiquitin protein ligase (BTRC) [29]. Moreover, which domain of ILF2 was bound by CREB and how the function of ILF2 was influenced by CREB need to be further investigated.

Taken together, our findings uncover a new phenomenon that the relationship between CREB and ILF2 promotes liver cancer growth, and ILF2 only stimulates CREB in the protein level. This interaction might be helpful to the diagnosis and treatment of liver cancer.

## Data Availability

The data used to support the findings of this study are available from the corresponding author upon request.

## Supplementary Materials

Supplementary Materials Supplementary Figure 1: endogenous ILF2 was immunoprecipitated by anti-ILF2 antibodies in Bel-7402 and SMMC-7721 cells, and the indicated proteins were measured by WB. Supplementary Figure 2: (a) the CREB and ILF2 mRNA levels were detected using qPCR in ten pairs of liver cancer and adjacent normal tissues. (b) Relationship between the CREB mRNA level and ILF2 level was measured. (c) The CREB and ILF2 protein expressions were detected using WB in ten pairs of liver cancer and adjacent normal tissues. (d) Liver cancer tissues and adjacent noncancerous tissues were IHC-stained using anti-IgG antibodies. Scale bar, 500 *μ*m. (e) ILF2-FLAG and CREB-HA were cotransfected into Bel-7402 cells as indicated. Exogenous tag proteins were detected by co-IP assays as indicated. (f) *In vitro* co-IP was performed by purified CREB and ILF2 proteins with anti-CREB or anti-ILF2 antibodies, followed by western blot using antibodies indicated. Supplementary Figure 3: (a, b) representative images for soft agar colony formation with indicated plasmids transfected. Scale bar, 500 *μ*m. (c, d) CREB and ILF2 expressions were measured by western blots with indicated plasmids transfected. Supplementary Figure 4: (a, b) mRNA levels of MCAM and HULC in control cells and Bel-7402 or SMMC-7721 cells with ILF2 knockdown, ILF2 overexpression, and ILF2 knockdown and ILF2 overexpression simultaneously were detected by qPCR. (c) Schematic representation of WT-, Del-pKID-, and Del-bZIP-CREB. (d) Lysates from Bel-7402 cells overexpressing indicated plasmids were immunoprecipitated by anti-HA antibodies, and coimmunoprecipitation of CREB-HA and ILF2-FLAG was measured by WB. (e) Lysates from Bel-7402 cells overexpressing indicated plasmids were immunoprecipitated by anti-FLAG antibodies, and coimmunoprecipitation of CREB-HA and ILF2-FLAG was measured by WB. (f) ILF2, CREB, and p-CREB protein levels were measured using WB with indicated plasmids transfected in Bel-7402 and SMMC-7721 cells.
