# Supplementary material for: ILF2 Directly Binds and Stabilizes CREB to Stimulate Malignant Phenotypes of Liver Cancer Cells
Source: Anal Cell Pathol (Amst). 2019 Feb 10;2019:1575031. doi: 10.1155/2019/1575031 (PMC6387701; doi:10.1155/2019/1575031)
Supplement: Supplementary Materials — Supplementary Figure 1: endogenous ILF2 was immunoprecipitated by anti-ILF2 antibodies in Bel-7402 and SMMC-7721 cells, and the indicated proteins were measured by WB. Supplementary Figure 2: (a) the CREB and ILF2 mRNA levels were detected using qPCR in ten pairs of liver cancer and adjacent normal tissues. (b) Relationship between the CREB mRNA level and ILF2 level was measured. (c) The CREB and ILF2 protein expressions were detected using WB in ten pairs of liver cancer and adjacent normal tissues. (d) Liver cancer tissues and adjacent noncancerous tissues were IHC-stained using anti-IgG antibodies. Scale bar, 500 μm. (e) ILF2-FLAG and CREB-HA were cotransfected into Bel-7402 cells as indicated. Exogenous tag proteins were detected by co-IP assays as indicated. (f) In vitro co-IP was performed by purified CREB and ILF2 proteins with anti-CREB or anti-ILF2 antibodies, followed by western blot using antibodies indicated. Supplementary Figure 3: (a, b) representative images for soft agar colony formation with indicated plasmids transfected. Scale bar, 500 μm. (c, d) CREB and ILF2 expressions were measured by western blots with indicated plasmids transfected. Supplementary Figure 4: (a, b) mRNA levels of MCAM and HULC in control cells and Bel-7402 or SMMC-7721 cells with ILF2 knockdown, ILF2 overexpression, and ILF2 knockdown and ILF2 overexpression simultaneously were detected by qPCR. (c) Schematic representation of WT-, Del-pKID-, and Del-bZIP-CREB. (d) Lysates from Bel-7402 cells overexpressing indicated plasmids were immunoprecipitated by anti-HA antibodies, and coimmunoprecipitation of CREB-HA and ILF2-FLAG was measured by WB. (e) Lysates from Bel-7402 cells overexpressing indicated plasmids were immunoprecipitated by anti-FLAG antibodies, and coimmunoprecipitation of CREB-HA and ILF2-FLAG was measured by WB. (f) ILF2, CREB, and p-CREB protein levels were measured using WB with indicated plasmids transfected in Bel-7402 and SMMC-7721 cells. [file 1575031.f1.docx]

Supplementary Figure 1


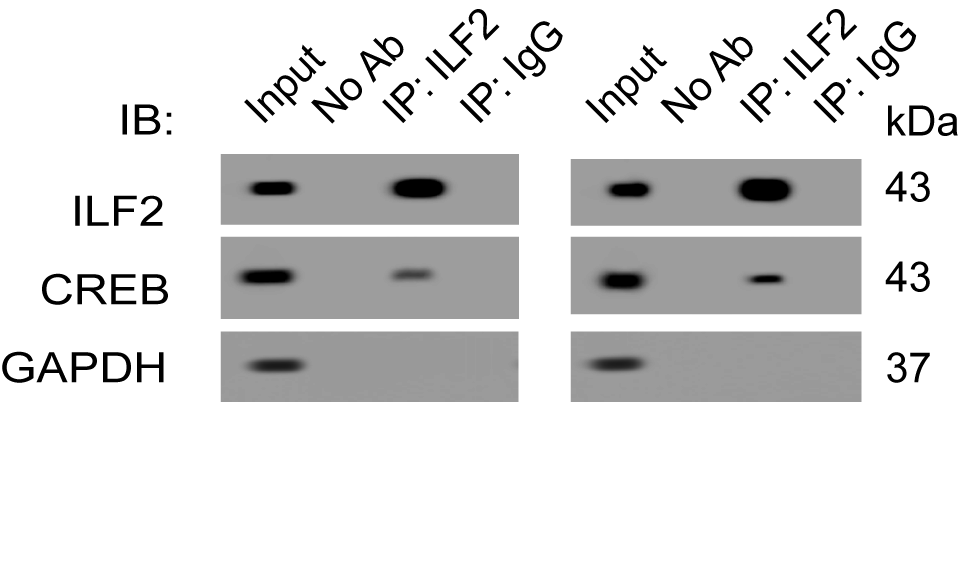


Endogenous ILF2 was immunoprecipitated by anti-ILF2 antibodies in Bel-7402 and SMMC-7721 cells, and the indicated proteins were measured by WB.

Supplementary Figure 2


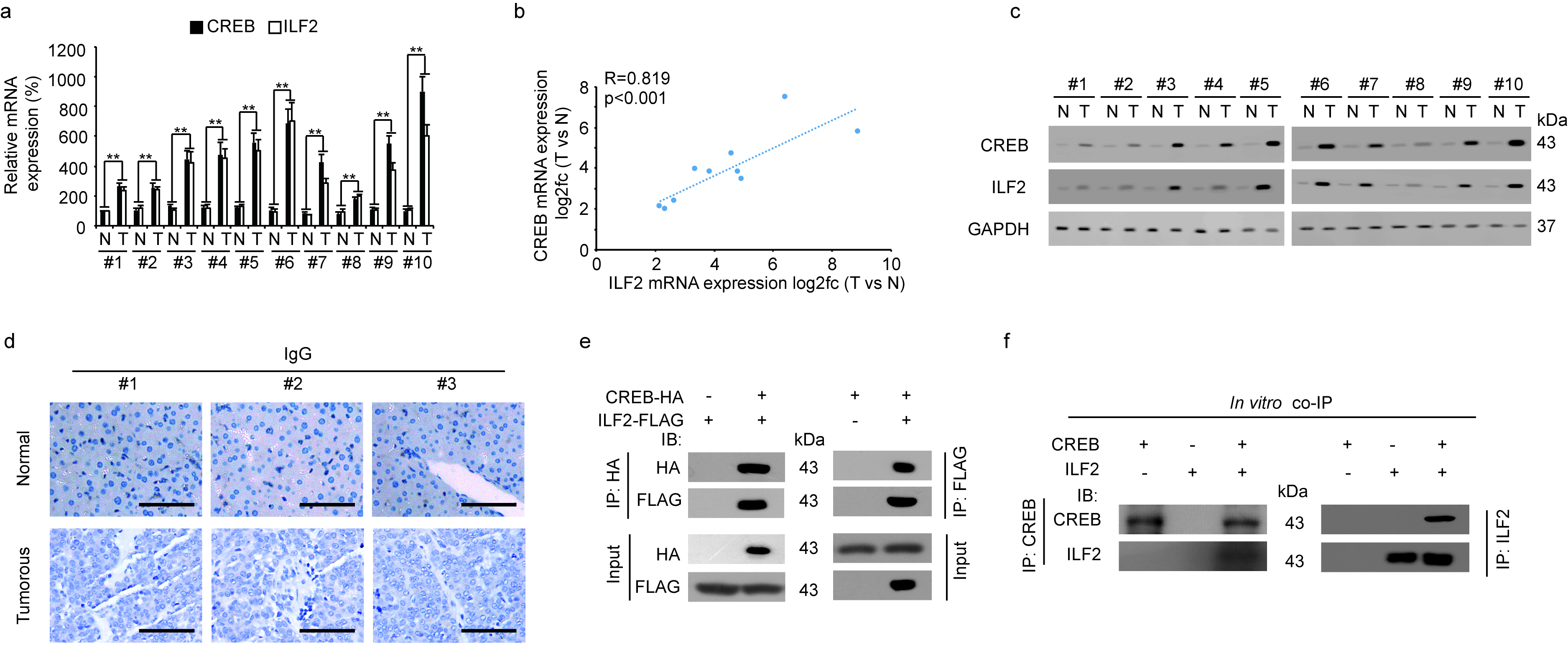


(a) The CREB and ILF2 mRNA levels were detected using qPCR in ten pairs of liver cancer and adjacent normal tissues.

(b) Relationship between the CREB mRNA level and ILF2 level was measured.

(c) The CREB and ILF2 protein expressions were detected using WB in ten pairs of liver cancer and adjacent normal tissues.

(d) Liver cancer tissues and adjacent non-cancerous tissues were IHC stained using anti-IgG antibodies. Scale bar, 500 μm.

(e) ILF2-FLAG and CREB-HA were co-transfected into Bel-7402 cells as indicated. Exogenous tag proteins were detected by co-IP assays as indicated. (f) *In vitro* Co-IP were performed by purified CREB and ILF2 proteins with anti-CREB or anti-ILF2 antibodies, followed by western blot using antibodies indicated.

Supplementary Figure 3


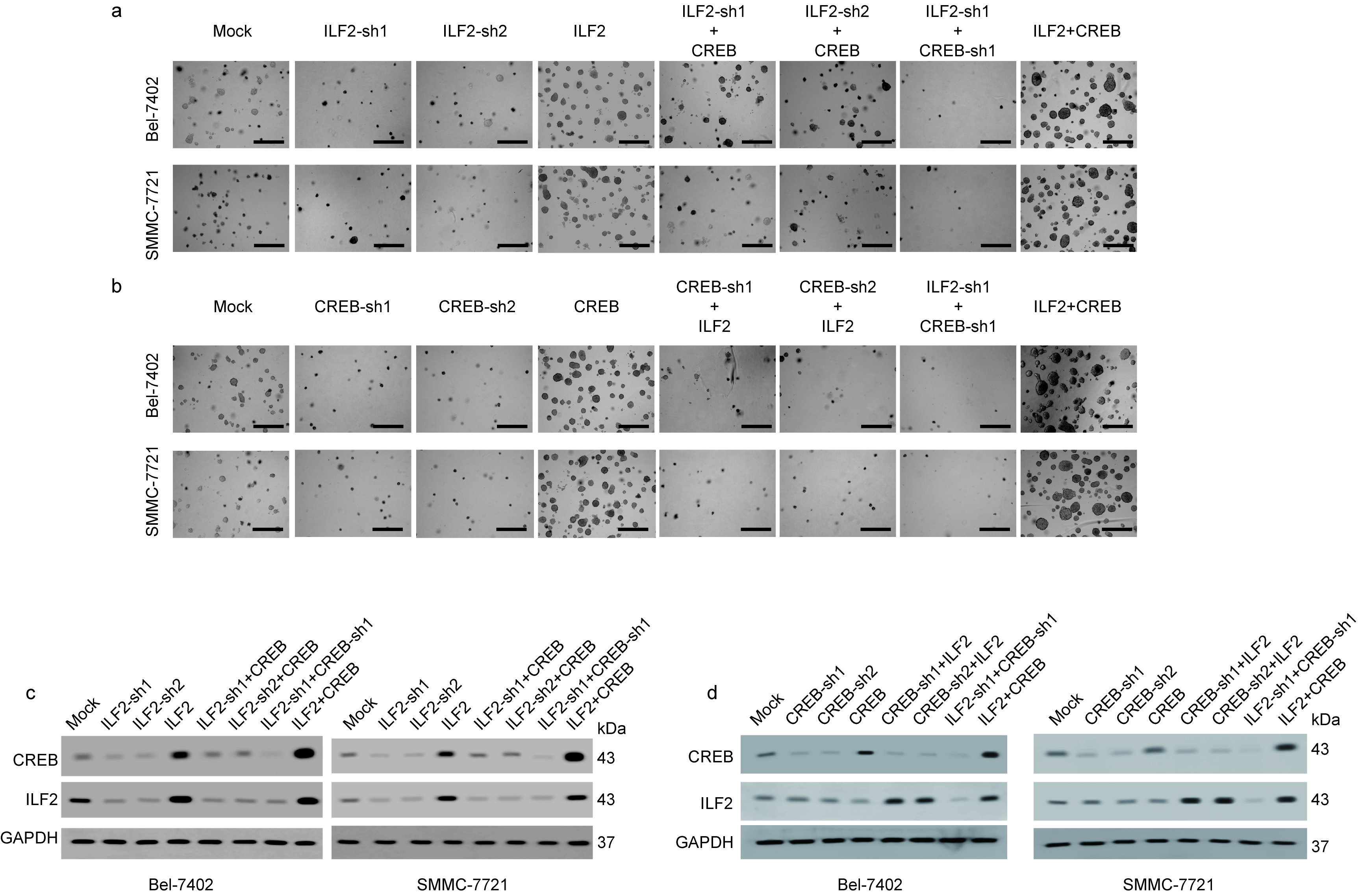


(a, b) Representative images for soft agar colony formation with indicated plasmids transfected. Scale bar, XX μm.

(c, d) CREB and ILF2 expressions were measured by Western blots with indicated plasmids transfected.

Supplementary Figure 4


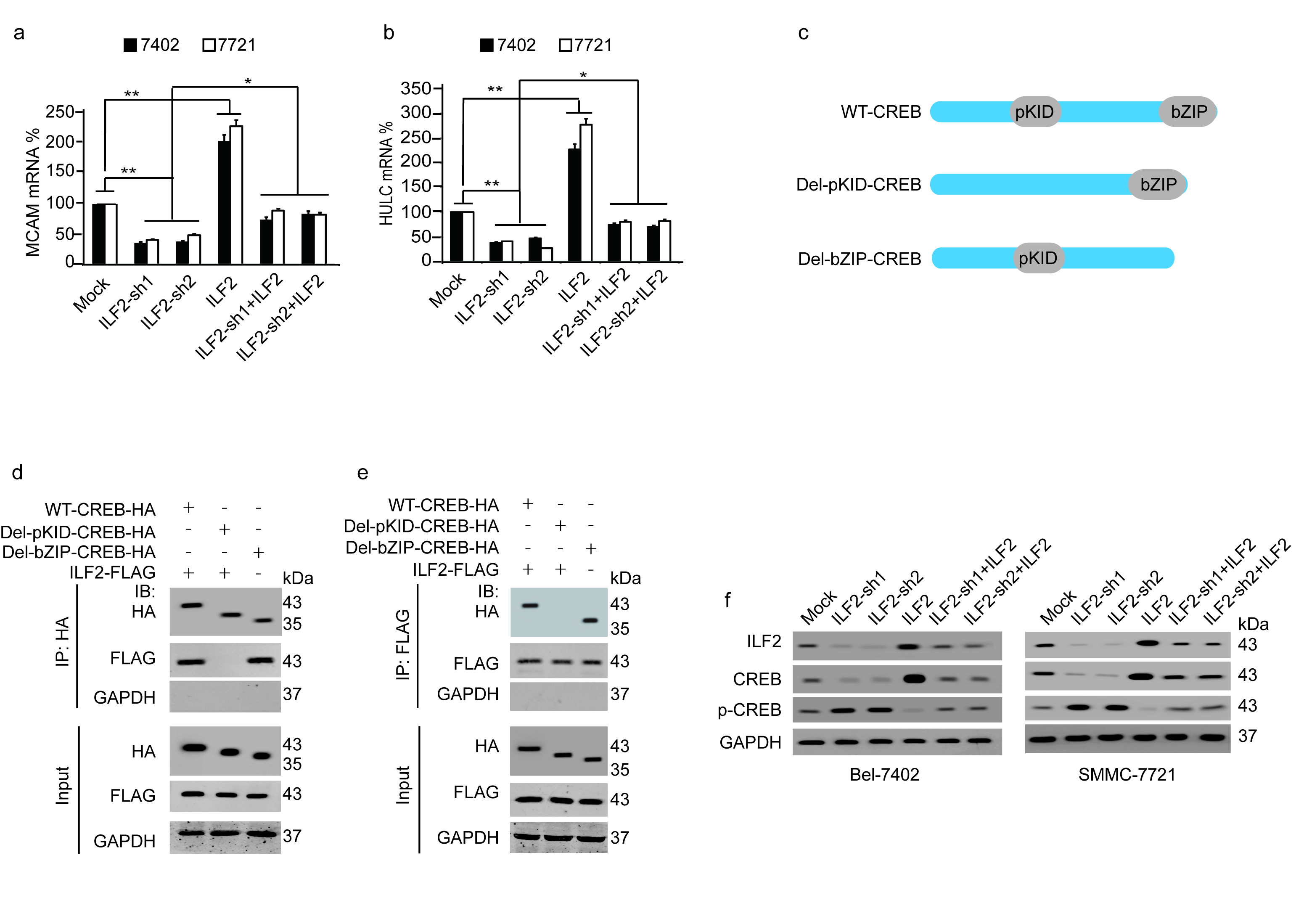


(a, b) mRNA levels of MCAM and HULC in control cells and Bel-7402 or SMMC-7721 cells with ILF2 knockdown, ILF2 overexpression, ILF2 knockdown and ILF2 overexpression simultaneously were detected by qPCR.

(c) Schematic representation of WT-, Del-pKID and Del-bZIP CREB.

(d) Lysates from Bel-7402 cells overexpressing indicated plasmids were immunoprecipitated by anti-HA antibodies, and co-immunoprecipitation of CREB-HA and ILF2-FLAG was measured by WB.

(e) Lysates from Bel-7402 cells overexpressing indicated plasmids were immunoprecipitated by anti-FLAG antibodies, and co-immunoprecipitation of CREB-HA and ILF2-FLAG was measured by WB.

(f) ILF2, CREB and p-CREB protein levels were measured using WB with indicated plasmids transfected in Bel-7402 and SMMC-7721 cells.
